# Cyclic Cushing’s Syndrome – A Diagnostic Challenge

**DOI:** 10.3389/fendo.2021.658429

**Published:** 2021-04-22

**Authors:** Renata Świątkowska-Stodulska, Agata Berlińska, Katarzyna Stefańska, Przemysław Kłosowski, Krzysztof Sworczak

**Affiliations:** ^1^ Department of Endocrinology and Internal Medicine, Faculty of Medicine, Medical University of Gdańsk, Gdańsk, Poland; ^2^ Department of Obstetrics, Faculty of Medicine, Medical University of Gdańsk, Gdańsk, Poland

**Keywords:** cyclic Cushing’s syndrome, cyclic hypercortisolemia, desmopressin test, hair cortisol, diagnosis of Cushing’s syndrome, DDAVP test

## Abstract

Cyclic Cushing’s syndrome (also known as intermittent or periodic) is a disease characterized by periods of transient hypercortisolemia shifting into periods of normo- and/or hypocortisolemia. Diagnosis of cyclic Cushing’s syndrome is based on at least three periods of confirmed hypercortisolemia interspersed by two periods of normocortisolemia. Cyclic Cushing’s syndrome is one of the greatest challenges in modern endocrinology due to its diverse clinical picture, unpredictable duration and frequency of phases, and various etiologies. We discuss a diagnostic algorithm for periodic hypercortisolemia with special regard to hair cortisol analysis and desmopressin stimulation test which both seem to be helpful in finding the correct answer.

## Introduction

Cyclic Cushing’s syndrome (also known as intermittent or periodic) is a disease characterized by periods of transient hypercortisolemia shifting into periods of normo- and/or hypocortisolemia. Just as classic Cushing’s syndrome, cyclic hypercortisolemia may arise from hormonal activity of corticotropinoma (approximately 80% of all cases), ectopic adrenocorticotropic hormone (ACTH; corticotropin) release, or ACTH-independent causes (1–9). Retrospective analysis of 201 patients with Cushing’s syndrome showed that 15% of them met the diagnostic criteria of intermittent hypercortisolemia and up to 70% showed evidence of cyclicity before the diagnosis (10). Some authors believe that cyclic Cushing’s syndrome might be more common than previously assumed. Giorgi et al. demonstrated periodic nature of subclinical hypercortisolemia in 18% patients with hormonally active adrenal incidentalomas (3). The fluctuation of observed abnormalities may explain the difficulties in diagnostics of periodic hypercortisolemia and often ambiguous results (3, 11). These dilemmas seem to be especially pronounced in ACTH-dependent cases (6, 7, 12).

Diagnosis of cyclic Cushing’s syndrome is based on at least three periods of confirmed hypercortisolemia interspersed by two periods of normocortisolemia (13, 14). Cyclic Cushing’s syndrome is one of the greatest challenges in modern endocrinology due to its diverse clinical picture, unpredictable duration and frequency of phases, and various etiologies. Patients may present with different severity of signs and symptoms appearing in either transient or continuous pattern. Sometimes only a few manifestations, such as recurrent peripheral edema, cardiac arrythmia, or hypokalemia, are present (15–17). Most commonly, the suspicion of cyclic Cushing’s syndrome arises in individuals suspected of hypercortisolemia but not meeting the full diagnostic criteria of any particular disease. Periodic hypercortisolemia should be also considered in patients whose initial tests confirm autonomic corticosteroid production but normocortisolemia follows. Duration of phases can range from 12 hours to 86 days as shown by available case reports; disease-free periods are unpredictable and they can hover from days to months (13, 18).

## Pathophysiology

Pathophysiology of cyclic hypercortisolemia may involve hypothalamic dysfunction with different degree of corticotroph cell response to neurotransmitters such as corticoliberin (CRH), dopamine, neuroepinephrine, serotonine and/or γ-aminobutyric acid (GABA). Other possible causes of intermittent signs and symptoms include spontaneous bleeding into the pituitary tumor followed by disrupted hormone synthesis in the neoplastic corticotroph cells, and persistent tumor response to the hypothalamus-pituitary-adrenal regulatory mechanisms (negative feedback) (5, 13, 15, 18, 19).

## Diagnostics of Endogenous Hypercortisolemia

**Figure 1:**
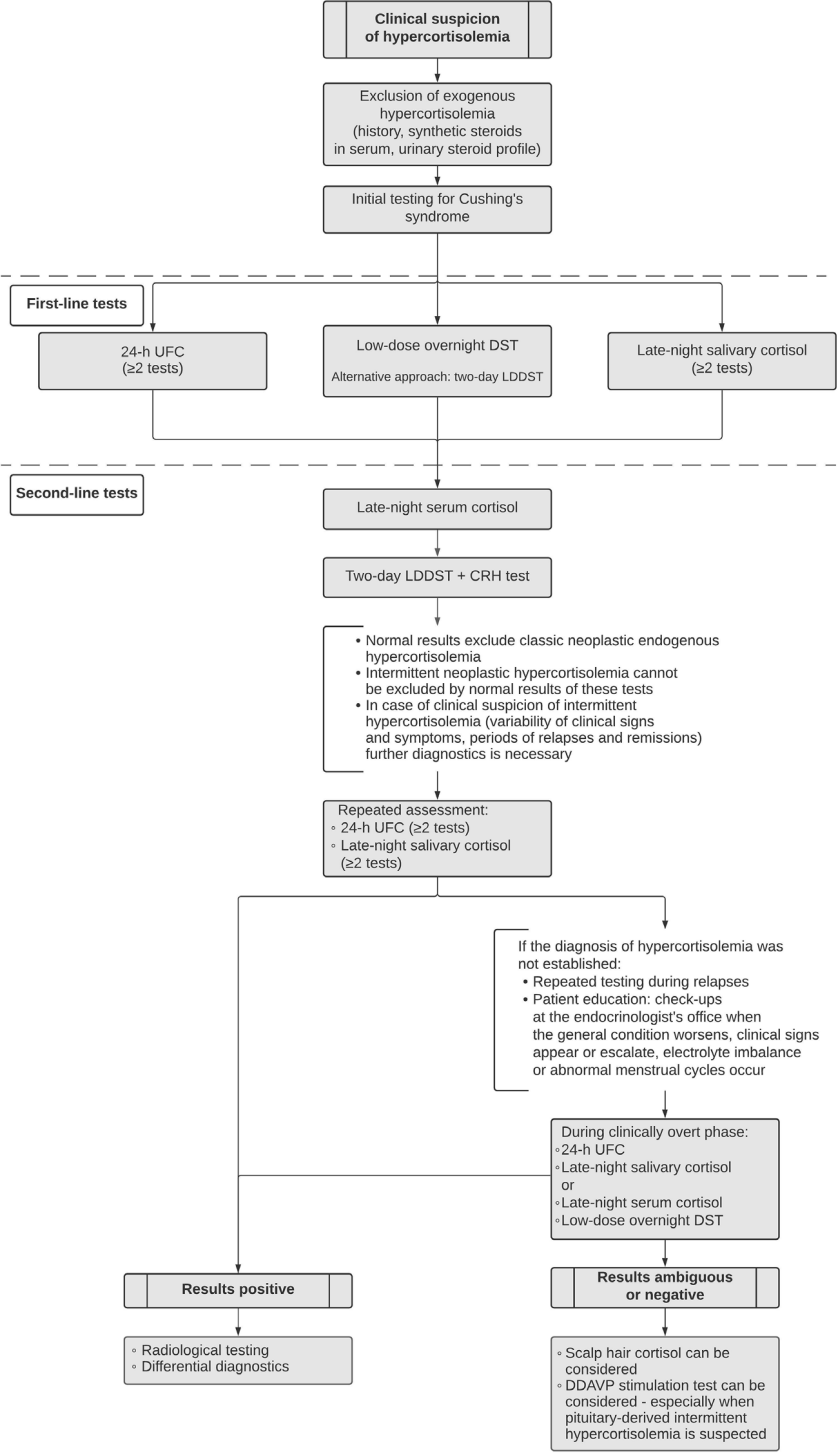
Proposed diagnostic algorithm for suspected cyclic Cushing’s syndrome.

Initial assessment of suspected endogenous hypercortisolemia includes first-line tests such as urinary free cortisol (UFC) – at least twice, late-night salivary cortisol – at least twice, and/or the low-dose overnight dexamethasone suppression test (DST). The Endocrine Society suggests further evaluation if at least one out of the aforementioned screening tests is positive (20). Certain scenarios necessitate the use of alternative initial tests such as late-night serum cortisol or two-day low-dose dexamethasone suppression test (LDDST). The latter one seems especially helpful due to improved specificity as compared with the overnight DST. If laboratory results are ambiguous, the second-line diagnostic approach may include LDDST with consecutive CRH administration (20, 21) (**Figure 1**). It is especially useful for distinguishing between Cushing’s syndrome and non-neoplastic hypercortisolemia (previously referred to as pseudo-Cushing’s syndrome). The cut-off values for all of the aforementioned tests are listed in the (**Table 1**).

**Table 1:** Diagnostic procedures commonly used in evaluation of hypercortisolemia.

Diagnostic procedure	Clinical use	Cutoff values
**First- and second-line screening tests for hypercortisolemia**		
24-h UFC	Suspicion of hypercortisolemia – screening test	Depends on laboratory and assay method
Low-dose overnight DST	Suspicion of hypercortisolemia – screening test	<50 nmol/l (1,8 µg/dl) in healthy subjects
Late-night salivary cortisol	Suspicion of hypercortisolemia – screening test	Cut-off values vary depending on population and assay method (20, 22–24)
Two-day LDDST	Suspicion of hypercortisolemia – screening test	<50 nmol/l (1,8 µg/dl) in healthy subjects
Late-night serum cortisol	Suspicion of hypercortisolemia	<50 nmol/l (1,8 µg/dl) in sleeping healthy subjects
Two-day LDDST with consecutive CRH test	Suspicion of hypercortisolemia, useful in non-neoplastic hypercortisolemia	Serum cortisol stays suppressed <38 nmol/l (1,4 µg/dl) after CRH administration in healthy subjects and non-neoplastic hypercortisolemia
DDAVP stimulation test	Suspicion of ACTH-dependent hypercortisolemia, useful in cyclic hypercortisolemia, useful in distinguishing between neoplastic and non-neoplastic hypercortisolemia (25)	Increase of plasma ACTH by at least 50% in ACTH-dependent cases
Hair cortisol	Suspicion of hypercortisolemia, useful in cyclic hypercortisolemia	Depends on laboratory and assay method
**Evaluation of origin of hypercortisolemia**		
Morning plasma ACTH	Evaluation of origin of hypercortisolemia (ACTH-dependent vs. ACTH-independent)	>20 pg/ml in ACTH-dependent cases <10 pg/ml in ACTH-independent cases
CRH stimulation test	Evaluation of origin of ACTH-dependent hypercortisolemia, useful in distinguishing between ACTH-dependent and ACTH-independent neoplastic hypercortisolemia when ACTH is indeterminate (10-20 pg/ml) (26)	Increase of plasma ACTH by 35-50% or serum cortisol by 14-20% in ACTH-dependent cases of pituitary origin
Two-day HDDST	Evaluation of origin of ACTH-dependent hypercortisolemia, *however its use remains controversial*	Drop of serum cortisol by at least 50% in corticotropinoma

DST, dexamethasone suppression test; LDDST, low-dose dexamethasone suppression test; HDDST, high-dose dexamethasone suppression test; ACTH, corticotropin; CRH, corticoliberin; DDAVP, desmopressin; UFC, urinary free cortisol.

Drug interactions should not be underestimated during the investigation. Some medications can alter dexamethasone metabolism by CYP3A4 induction or inhibition. Drugs such as for instance phenytoin, phenobarbital, carbamazepine, and rifampicine are known as cytochrome inducers, while itraconazole, fluoxetine, diltiazem, and ritonavir are examples of inhibitors. Circulating transcortin (CBG) concentration increases due to estrogen or mitotane use. Various xenobiotics can intensify cortisuria – notable examples are carbamazepine and fenofibrate.

Differential diagnostics of the origin of hypercortisolemia should follow after at least two initial tests come back positive since none of the screening tests alone is sensitive and specific enough to confirm endogenous hypercortisolemia. Differentiation of the origin of hypercortisolemia can be based on morning plasma ACTH, and CRH stimulation test. Sometimes – though nowadays it remains controversial – two-day high-dose dexamethasone suppression test (HDDDST) may be used (20, 21, 27). Interpretations of different tests can be found in **Table 1**. If no apparent hypophyseal mass is found in magnetic resonance and pituitary-derived ACTH-dependent hypercortisolemia is suspected, cavernous sinus catheterization can be considered.

Adrenal imaging is necessary whenever ACTH-independent hypercortisolemia is suspected.

## Distinct Features in Diagnostics of Cyclic Hypercortisolemia

Whenever cyclic hypercortisolemia is suspected, it is advised to start the investigation with UFC and/or late-night salivary cortisol (5, 13, 20, 28). If the initial results are within the normal limits but clinical suspicion remains strong, evaluation should be repeated for months, if not years, depending on the severity of presented symptoms. The low-dose overnight DST and two-day LDDST are generally contraindicated during remission since subjects can achieve full suppression. Importantly, hormonal suppression tests during relapses may generate paradoxical responses resulting in peaks of serum cortisol (15, 29).

Differential diagnosis of cyclic Cushing’s syndrome should include: exogenous steroid use, mild autonomous hypercortisolemia (previously referred to as subclinical Cushing’s syndrome), non-neoplastic hypercortisolemia, use of xenobiotics affecting hormonal tests, glucocorticoid resistance syndrome, and factitious disorder. It is crucial to continuously repeat biochemical testing to finally confirm the diagnosis (13). The chance of accurate diagnosis is the highest during the active phase or shortly after it concludes. According to the Endocrine Society, excessive exposure to exogenous steroids and iatrogenic Cushing’s syndrome should be excluded before the proper diagnostics starts (20). Some characteristics typical for Cushing’s syndrome such as menstrual irregularity, acne, and hirsutism, overlap with features of polycystic ovary syndrome (PCOS), insulin insensitivity, obesity or late-onset congenital adrenal hyperplasia. If there are any doubts regarding the origin of observed abnormalities, initial testing for Cushing’s syndrome can be introduced. In obesity and PCOS the low-dose overnight DST and/or late-night serum cortisol are the tests of choice (30, 31).

Rare but possible explanation of apparent intermittent hypercortisolemia includes factitious disorder. Factitious disorder, also known as Münchhausen’s syndrome, is a psychiatric disorder resulting in patients deliberately fabricating signs and symptoms of an illness. Descriptions of factitious Cushing’s syndrome are infrequent and cases involve the use of various exogenous corticosteroids (32, 33). Steroids can be either conventionally used as drugs or added to already collected specimens (for example urine). Suppressed ACTH and DHEA-S levels may suggest exogenous steroid use. Professional laboratories sometimes offer synthetic glucocorticoid serum analysis. Exogenous steroids might be cross-reactive with endogenous cortisol, therefore specific assays discerning their presence should be used. Urine samples can be assessed using high-performance liquid chromatography (33) and/or gas chromatography-mass spectrometry which proved to be useful in tracking a variety of steroids, and cortisol breakdown products can be measured in urine as well (13). As fatal cases of factitious hypercortisolemia were reported (32), patients suspected of Münchhausen’s syndrome should be promptly examined by an experienced psychiatrist and appropriate assistance should be offered to them.

Well-established factors provoking non-neoplastic hypercortisolemia are alcohol abuse, poorly controlled diabetes mellitus, severe depression, obesity, and pregnancy (34). These conditions result in excessive activation of the hypothalamus-pituitary-adrenal (HPA) axis without autonomous hypercortisolemia. Even though observed abnormalities can closely resemble those arising from neoplastic hypercortisolemia, most signs and symptoms should resolve after the underlying condition fades (20, 21).

Identification of the origin of hypercortisolemia in cyclic Cushing’s syndrome is based on the same tests as in the classic form of the disease: morning plasma ACTH, CRH stimulation test, rarely HDDST (**Table 1**). Just like in the classic Cushing’s disease, if no apparent hypophyseal mass is found in magnetic resonance and pituitary-derived ACTH-dependent hypercortisolemia is suspected, cavernous sinus catheterization can be considered. If intermittent hypercortisolemia is suspected, the procedure should be performed during active phase of the disease. Otherwise, the results might come back negative with the patient unnecessarily exposed to an invasive procedure (1, 19).

## Miscellaneous Procedures

Since diagnostics of intermittent hypercortisolemia can carry distinct difficulties, some experts suggest using less common procedures (35–44).

Over the past few years, scalp hair cortisol measurement was gaining an ever-growing attention. The assessment of hair cortisol can be especially helpful in identification of cyclic Cushing’s syndrome. Average hair growth oscillates around 1 cm per month, which allows retrospective tracking of varying concentrations of circulating cortisol. Elevated concentration of hair cortisol in patients with endogenous hypercortisolemia was demonstrated on multiple occasions and can be therefore seen as a marker of exposition to systemic cortisol (35–40). More so, direct relationship between hair cortisol and disease activity was proven in Cushing’s syndrome (38). Hair cortisol is so far the only method granting retrospective analysis of exposition to cortisol over the past months or years (depending on the hair length). Henceforth, it seems to be a highly helpful tool in validating periodic cortisol overproduction. Sample collection is simple, noninvasive, there is no need to store the hair in any special environment and the specimen can be transported to the testing laboratory *via* standard mail. Hair sample should be obtained from the occipital region and cut as close to the skin as possible. Next, the hair should be stored in an envelope or a plastic bag in a cold, dark place. Hair cortisol concentration remains stable for months in room temperature. So far, multiple methods of preparing the hair and its analysis were published; their use varies depending on the laboratory. Enzyme immunoassay (EIA) and liquid chromatography tandem mass spectrometry (LC-MS) are among the most common techniques (36, 40).

Desmopressin (DDAVP) stimulation test is another helpful method in evaluation of cyclic Cushing’s syndrome (41). Currently, it is not recommended in routine testing because uniform criteria of interpretation are still to be developed (21). Desmopressin stimulation test relies on measuring plasma ACTH concentration before and 10, 20, 30 minutes after intravenous administration of 10 µg desmopressin. Significant rise in ACTH concentration should occur in pituitary ACTH-dependent Cushing’s syndrome, while it should not be observed in healthy individuals, non-neoplastic hypercortisolemia, ACTH-independent hypercortisolemia, and ectopic ACTH production (42–49). The aforementioned response to DDAVP is a result of V3 receptor overexpression in corticotroph neoplastic cells. Normal pituitary cells typically exhibit minimal or nonexistent response to desmopressin. The test might be used not only to establish the diagnosis, but to assess potential relapses – even as a promising early marker of long-term results of surgery (50, 51). Data showing typical response to desmopressin in ACTH-dependent Cushing’s syndrome in a patient with intermittent hypercortisolemia is available and in that case testing was performed during remission (43). DDAVP stimulation test may be taken into consideration in cases of pituitary-derived cyclic hypercortisolemia presenting with negative or ambiguous results of conventional tests. More so, the DDAVP test may be suitable for distinguishing between neoplastic and non-neoplastic hypercortisolemia due to the pathophysiology of observed reaction (25, 51).

Both hair cortisol and DDAVP stimulation test are not routinely used in differential diagnostics of hypercortisolemia. However, periodic Cushing’s syndrome is a clinical challenge often defying routine testing and therefore asks for alternative options. We would like to propose a diagnostic algorithm for cyclic Cushing’s syndrome as shown in **Figure 1**.

## Treatment

Once the diagnosis is finally confirmed, patients with cyclic Cushing’s syndrome should follow conventional treatment suited for patients with endogenous hypercortisolemia. Surgery remains the most effective and preferable method of treatment in most hormonally active adenohypophyseal masses (with prolactinoma being an exception). Unfortunately, complete resection in clinically advanced disease might not be achievable. It is especially important for the patients to be cared for in experienced neurosurgical centers as it was proven to improve the outcomes (52). If resection is not complete or unobtainable at all, radiotherapy and/or continuous pharmacotherapy should be introduced. Decision regarding appropriate type of intervention should be discussed in a multidisciplinary medical team to provide personalized treatment.

Radiotherapy is a valuable second-line treatment in patients not meeting the criteria of full excision or with recurrent Cushing’s disease. The most prevalent techniques are stereotactic radiosurgery (SRS) and fractionated external beam radiation therapy (EBRT) (53, 54). Qualification for radiotherapy should be performed by an experienced radiation oncologist to ensure that the chosen method is optimal for the patient. One of the main concerns is preserving the optic chiasm. After the treatment, the patient should be carefully evaluated for potential side effects, such as permanent neurological damage or hypopituitarism.

Currently, a vast choice of drugs is available: steroidogenesis inhibitors [metyrapone, ketoconazole, mitotane, etomidate, or recently FDA-approved orphan drug osilodrostat (55)], glucocorticoid receptor-directed agents (mifepristone), and pituitary-directed agents (cabergoline, pasireotide). If the condition is especially severe and/or recurrent, bilateral adrenalectomy remains a potentially life-saving option (20). As signs and symptoms of Cushing’s syndrome tend to progress over time and the disease can eventually become fatal, appropriate treatment should not be postponed. After treatment, all patients should be screened for hormone deficiencies, as well as for signs and symptoms of a relapse.

Surgery remains the recommended modality of treatment in cyclic Cushing’s syndrome arising from ectopic ACTH-secreting tumors, or hormonally active adrenal tumors (unilateral adrenalectomy).

## Summary

Cyclic Cushing’s syndrome usually causes significant diagnostic problems. The diagnosis should be taken into consideration whenever clinical suspicion of hypercortisolemia meets normal results of hormonal tests. In that scenario, the work-up should be repeated, especially when clinical signs and symptoms (re)appear. Increased 24-h UFC, elevated late-night salivary and/or serum cortisol can confirm cyclic hypercortisolemia. DST may provoke paradoxical rise of serum cortisol in relapsing patients. In questionable and ambiguous cases, hair cortisol and DDAVP stimulation test should be kept in mind as a valuable option.

## Author Contributions

RŚ-S, AB, KaS: These authors contributed equally to this work and share first authorship: conceived the idea of the work, contributed to the design of publication and reference collection, and were responsible for preparing the manuscript. PK: involved in preparing the manuscript and reference collection. KS: proof-reading and revision of the manuscript. All authors contributed to the article and approved the submitted version.

## Conflict of Interest

The authors declare that the research was conducted in the absence of any commercial or financial relationships that could be construed as a potential conflict of interest.
